# Sex- and site-specific reference data for size-invariant properties using multi-stack HRpQCT

**DOI:** 10.1093/jbmrpl/ziag077

**Published:** 2026-04-25

**Authors:** Simone Poncioni, Dominique Lüscher, Michael Indermaur, Daniela A Frauchiger, Christian Meier, Philippe Zysset, Kurt Lippuner

**Affiliations:** ARTORG Center for Biomedical Engineering Research, University of Bern, 3010 Bern, Switzerland; Department of Osteoporosis, Bern University Hospital, 3010 Bern, Switzerland; ARTORG Center for Biomedical Engineering Research, University of Bern, 3010 Bern, Switzerland; ARTORG Center for Biomedical Engineering Research, University of Bern, 3010 Bern, Switzerland; ARTORG Center for Biomedical Engineering Research, University of Bern, 3010 Bern, Switzerland; Department of Osteoporosis, Bern University Hospital, 3010 Bern, Switzerland; Division of Endocrinology, University Hospital Basel, 4031 Basel, Switzerland; ARTORG Center for Biomedical Engineering Research, University of Bern, 3010 Bern, Switzerland; ARTORG Center for Biomedical Engineering Research, University of Bern, 3010 Bern, Switzerland; Department of Osteoporosis, Bern University Hospital, 3010 Bern, Switzerland

**Keywords:** bone strength, distal radius, distal tibia, finite element analysis, HRpQCT, osteoporosis

## Abstract

HRpQCT is emerging as a promising evolution to DXA for longitudinal assessment of bone properties and strength estimation beyond FN aBMD, as it provides a detailed 3D representation and separate quantification of trabecular and cortical compartments. Reference data exist for thin single stacks of 10*.*2 mm in second-generation HRpQCT, but these sections may not fully capture clinically relevant fracture locations and pose challenges for longitudinal monitoring due to their limited thickness. Reported parameters are mainly size-dependent properties susceptible to bias from skeletal dimensions, potentially concealing changes in bone quality at the material level. Moreover, microstructural parameters are derived from densitometric information, making them partially redundant. This study provides the first age-, sex-, and site-specific reference data for a novel multi-stack on second-generation HRpQCT at the distal radius and tibia in 381 healthy participants (144F, 237M) from a primarily Caucasian population aged 20-92 yr and identifies the size-independent parameters most sensitive to age for improved bone health assessment. Six size-independent parameters relevant for estimated mechanical properties or exhibiting short trend assessment intervals were selected as candidates for improved bone health assessment: 2 densitometric properties (total volumetric BMD, cortical volumetric BMD), 1 size-independent geometrical property (relative cortical thickness), 2 microstructural properties (trabecular degree of anisotropy, trabecular bone volume over total volume), and 1 mechanical property (apparent yield stress [^app^*σ*_y_]) estimated by homogenized finite elements. Intensive mechanical properties provided more sensitive follow-up estimations. Cortical volumetric BMD, especially in the weight-bearing tibia in women, was the most sensitive with age. Matched comparisons with single-stack counterparts demonstrated good agreement between densitometric and microstructural properties, supporting potential cross-study and cross-protocol comparisons. The present work proposes an alternative set of size-independent variables for multi-stack HRpQCT, which may offer a refined assessment of bone health and longitudinal monitoring.

## Graphical Abstract

**Graphical Abstract:**
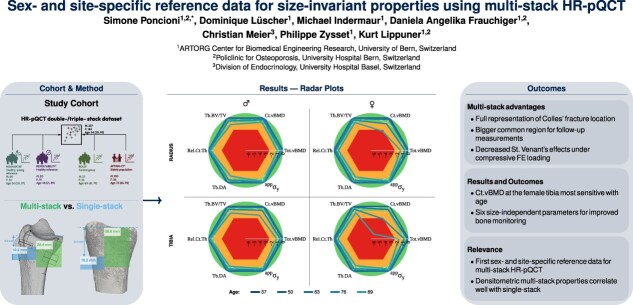

## Introduction

Osteoporosis is characterized by a loss of bone density and micro-architectural deterioration, resulting in decreased bone strength, increased bone fragility, and susceptibility to fracture.^1^ The gold standard for the clinical diagnosis of osteoporosis includes lumbar spine, total, and FN areal BMD (aBMD) (gcm^−2^) measured by DXA.^2^ Although DXA-derived aBMD is a significant predictor of osteoporotic fractures,^3^ both its low resolution and the lack of 3D representation impose certain limitations when used for the estimation of fracture risk at the individual level.

HRpQCT has emerged as a valuable evolution to DXA for assessing distal skeletal bone health. This is attributed to its higher sensitivity to microstructural changes with respect to DXA,^4^ a higher resolution of 90 μm at 10% modulation transfer function (MTF) and an isotropic voxel size of 60.7 μm that allow the discrimination between cortical and trabecular compartments, and a low effective radiation dose. Moreover, advanced computational methods enable rapid assessment of bone structural mechanics from imaging data using homogenized finite elements (hFE).^5^^,^^6^ This technique provides an estimate of failure load, which is a more direct surrogate for bone strength than densitometric properties alone.^7^ The hFE model takes into account both density and microstructure, and is accomplished in a clinically efficient timeframe. What once required several hours to solve^6^ can now be evaluated on a standard workstation in ~15 min.^8^ HRpQCT is particularly well-suited for elucidating the microstructural mechanisms underlying fragility fractures by providing refined insights beyond aBMD, and is especially informative in discriminating fracture risk for patients where treatment decisions require careful consideration.^9^ Additionally, the higher sensitivity to longitudinal changes of HRpQCT in comparison with DXA renders it a suitable complementary diagnostic tool for treatment monitoring and cessation.^10^

Normative data were reported by Burt et al.^11^ for first-generation HRpQCT, and by Whittier et al.^12^ and Warden et al.^13^ for second-generation HRpQCT (XTremeCT I and II, Scanco Medical AG, Brüttisellen, Switzerland). Both studies were performed by scanning a single clinical stack (ie, a thin image of ∼10 mm of scanning height along the metaphysis). However, this standard HRpQCT clinical section may not fully capture clinically relevant regions affected by fractures, particularly at the distal radius. Whilst the standard stack positioning was designed to capture both cortical and trabecular compartments simultaneously, an extended ROI offers additional advantages, including broader coverage of fracture-prone sites such as the distal radius exposed to typical Colles’ fractures,^14^ and improved multi-stack imaging could facilitate longitudinal observations, through a substantially increased largest common height (LCH). To address these challenges, Schenk et al.^15^ implemented a modified acquisition protocol to optimize coverage while maintaining comparability with established methodologies. The ROI was doubled to 20*.*4 mm and tripled to 30*.*6 mm at the distal radius and the distal tibia, respectively.

To date, research has not yet determined age-, sex-, and site-specific reference data for multi-stack second-generation HRpQCT at the distal radius and tibia. Data acquired using the multi-stack protocol may not be directly comparable with findings on single-stack images, emphasizing the need for dedicated reference values and a direct comparison. Therefore, the first objective of this study is to collect the first large dataset with this protocol to develop age-, sex-, and site-specific reference data for multi-stack second-generation HRpQCT, and to evaluate the comparability of the two ROIs on the standard clinical output. Furthermore, HRpQCT has demonstrated considerable potential due to its higher sensitivity to longitudinal changes in comparison with DXA for follow-up measurements. However, most of the parameters proposed by the scanner manufacturer are derived from bone density or bone volume fraction (BV/TV) and are, therefore, partially redundant. Moreover, the size dependency of properties such as cortical perimeter (Ct.Pm) or F_y_ may interfere with sensitivity to longitudinal changes, potentially concealing clinically relevant skeletal changes. Consequently, the second objective is to explore which size-independent parameters demonstrate the highest sensitivity with age in our cohort and are thus more suitable as output candidates for improved distal bone health assessment.

## Materials and methods

This study evaluated bone health in a healthy Swiss cohort from 2017 to 2024 using HRpQCT, DXA, and standardized clinical assessments. The study population underwent multi-stack HRpQCT scans at both the distal radius and tibia, as well as a DXA scan at the FN. All measurements were conducted using a second-generation HRpQCT (XtremeCT II, Scanco Medical AG, Brüttisellen, Switzerland) and 2 densitometers (Discovery C, Hologic, Marlborough, MA, USA). In addition to imaging, participants completed a questionnaire covering demographic information and medical history. Both medical history and DXA-derived information were used to assess the probability of fracture occurrence using the fracture risk assessment tool (FRAX).^16^ Individuals with coexisting metabolic bone disease, a history of osteoporosis, or medical conditions affecting bone health were excluded from participation. Stiffness (S) and F_y_ were estimated with a recently developed hFE methodology.^8^

### Cohort

**Figure 1:**
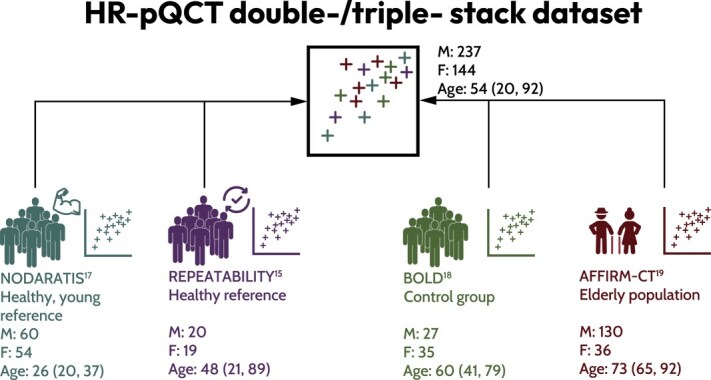
Study population that underwent double- and triple-stack HRpQCT scans at the distal radius and tibia, as well as DXA scans at the FN. Only healthy subjects without a history of osteoporotic fractures or any medical conditions affecting bone metabolism were included.^15^^,^^17–19^

Figure 1 provides a graphical summary of the study cohort. The study population consisted of 381 individuals (38% females) aged 20-92 yr, who were recruited from 4 different studies, each scanned at a single time point. Tables A4 and A5 in the Supplementary material summarize the demographics of the study population, including age, FN aBMD, BMI, and FRAX scores for major osteoporotic and hip fracture risk. The population was stratified into a younger group (20-39 yr) and an older group (40-99 yr). Statistical comparisons were conducted using the Student’s *t*-test for normally distributed variables (mean ± SD) and the Wilcoxon-Mann-Whitney test for non-normally distributed variables (median [IQR]). The participants of the first cohort were selected to establish a normative database of young, healthy individuals representative of the Swiss population (F = 54, M = 60, aged 20-37 yr), as outlined by Stuck et al.^17^ The second cohort—a subset of participants recruited under the same ethical application as the first—was recruited to evaluate the short-term repeatability error of HRpQCT outcomes (F = 19, M = 20, aged 21-92 yr), as described by Schenk et al.^15^ The participants of the third cohort were the non-diabetic control group part of a single-center, cross-sectional, case-controlled study on long-standing type 1 diabetes (F = 35, M = 27, aged 41-79 yr), conducted by Sewing et al.^18^ The fourth study included community-dwelling adults aged 65 or older from the study “A Fragility Fracture Integrative Risk Method for CT Recycling” (AFFIRM-CT). This prospective observational study was conducted to identify factors for fall and fragility fracture risk prediction^19^ (F = 36, M = 130, aged 65-92 yr). Given the complexity of defining the characteristics of healthy life in older individuals of the latter study population, more specific exclusion criteria were implemented to ensure a clearer delineation: a prior hip fracture, life expectancy of <1 yr, being bedridden or in a wheelchair, living in a nursing home, suffering from a bone pathology, or cognitive impairment. The first 2 studies were conducted at the University Hospital Bern under the approval of the ethics committee of the canton of Bern, Switzerland (BASEC ID: 2017-00882). The third study was conducted at the Endocrine Clinic of University Hospital Basel, Switzerland, and the University Hospital Bern, Switzerland, under the approval of the Northwest and Central Switzerland ethics committee (BASEC ID: 2018-01517). The measurements of the fourth study were performed at the University Hospital of Bern, Switzerland, and were approved by the local ethics committees of Bern and Geneva (BASEC ID: 2019-01327). All participants provided written informed consent.

### Image acquisition and processing

**Figure 2:**
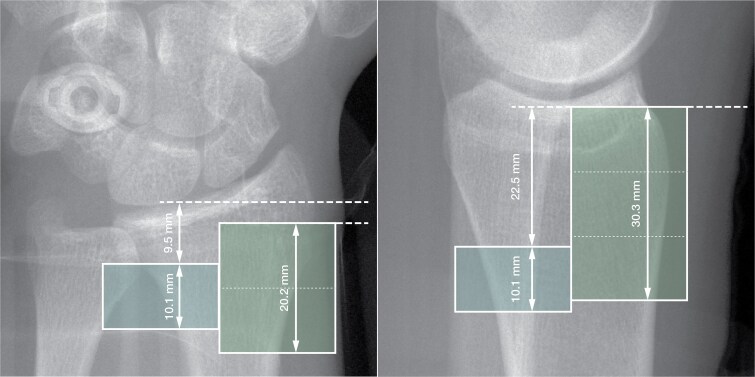
Scout view showing representative locations of the standardized single-stack protocol (left ROI) described by Bonaretti et al.^20^ and the multi-stack protocols proposed by Stuck et al.^17^ for image acquisition at the distal radius and tibia. (a) At the distal radius, the double-stack protocol (right ROI) fully encompasses the region of interest defined by the single-stack protocol. (b) At the distal tibia, the triple-stack protocol (right ROI) only partially overlaps with the region of interest of the single-stack protocol (left ROI).

HRpQCT imaging was performed on the non-dominant limb—or the non-fractured side in cases of a previous fracture—using a modified acquisition protocol. This imaging approach followed the standard clinical workflow, except for the number and positioning of the stacks. Images were acquired at a voxel size of 60*.*7 μm using the standard imaging settings (*U_p_* = 68 kV, *I* = 1460 μA, integration time *t* = 40 ms). At the radius, the ROI originated at the articular surface between the scaphoid and lunate fossae of the radiocarpal joint, extending for 20*.*4 mm (336 slices) to ensure coverage of the entire region affected by Colles’ fractures.^14^ At the tibia, the ROI started at the apex of the distal articular plateau and extended for 30*.*6 mm (504 slices), encompassing the epiphysis and metaphysis of the distal tibia. A comparison of the standard and modified ROIs is provided in Figure 2. During image acquisition, the limb was immobilized using the manufacturer’s cast to prevent involuntary motion. HRpQCT scans were evaluated for motion artifacts by trained operators following the criteria of Pialat et al.^21^ Images were rated on the proposed scale (1: no motion, 5: significant discontinuities in the cortical shell), and those graded above 3 were excluded. Image processing adhered to the manufacturer’s standard workflow (IPL Scanco Module 64-bit Version V5.16/FE-v02.02) to ensure comparability with prior studies. The periosteal contour was generated using a semi-automatic contouring algorithm and was verified by a single trained operator. Subsequently, the cortical and trabecular compartments were segmented using Gauss filtering (*σ* = 0*.*8, support = 1 voxel), coupled with a dual thresholding operation (320 $\frac{\mathrm{m}\mathrm{gHA}}{\mathrm{c}{\mathrm{m}}^3}$ on the trabecular compartment, 450 $\frac{\mathrm{m}\mathrm{gHA}}{\mathrm{c}{\mathrm{m}}^3}$ on the cortical compartment). Further details can be found in the description provided by Stuck et al.^17^ The standard clinical evaluation was then conducted to obtain geometrical, densitometric, and microstructural information. These adaptations allowed for a bigger ROI in clinically relevant bone sections while maintaining methodological consistency with prior research.

### hFE to assess mechanical behavior

The estimated mechanical behavior of the distal radius and tibia under uniaxial compressive load was simulated with hFE. Accurate mechanical assessment requires distinct modeling approaches for trabecular and cortical compartments, considering their unique structural and material properties. The thin cortex entails a more precise geometrical representation, which coarse voxel-based meshes cannot represent. Therefore, we employed a non-linear hFE methodology that accurately predicts both S and F_y_ using smooth structured meshes, as described in a recent study.^8^ Briefly, smooth, structured, hexahedral meshes were generated from a third-order BSpline representation of the periosteal and endosteal contours. Structural densities *ρ* were homogenized based on BV/TV in the trabecular compartment and on cortical volumetric BMD (Ct.vBMD) in the cortical compartment. Anisotropic structural properties were assigned using second-order, positive-definite fabric tensors. The fabric tensor in the trabecular compartment was calculated using the mean surface length (MSL), and transverse isotropy projected along the transverse axis was assigned to the cortical compartment. The constitutive model incorporated linear elasticity, yielding, and damage accumulation, resulting in irreversible strains.^22^ A fabric-based orthotropic model was fitted with elasticity properties for both compartments based on experimental literature^23^^,^^24^: trabecular (elastic modulus ${\mathrm{\varepsilon}}_0=10\ 480$ MPa, Poisson’s ratio ${\mathrm{\nu}}_0=0.2289$, shear modulus ${\mathrm{\mu}}_0=3350$ MPa, *k* = 1*.*55, *l* = 0*.*82), and cortical (${\mathrm{\varepsilon}}_0=15\ 992$ MPa, ${\mathrm{\nu}}_0=0.339$, ${\mathrm{\mu}}_0=5846$ MPa, *k* = 1*.*0, *l* = 1*.*0). Quadric yield constants in agreement with literature^25^ were specified for the trabecular compartment (maximum tensile strength ${\mathrm{\sigma}}_0^{+}=62.01$ MPa, maximum compressive strength ${\mathrm{\sigma}}_0^{-}=78.58$ MPa, maximum shear strength ${\mathrm{\tau}}_0=31.83$ MPa), and for the cortical compartment (${\sigma}_0^{+}=71.00$ MPa, ${\sigma}_0^{-}=124.20$ MPa, ${\tau}_0=41.30$ MPa).

Uniaxial monotonic compression simulations were performed using Abaqus (Abaqus 6.24-1, Simulia, Dassault Systèmes, Paris, France). The proximal nodes were encastred (*u* = 0), and the distal nodes were kinematically coupled to a reference point (RP) positioned at the center of inertia of the most distal surface. A uniform axial displacement of −1% strain (*u*) was applied with a linear ramp to the RP. The reaction forces and displacements were recorded along the longitudinal axis. The simulations were performed on 16 cores (AMD EPYC 7742 Processor, CPU maximum clock speed = 2*.*25GHz) using the Rocky Linux 9.3 Blue Onyx operating system. Extensive (structural) properties, namely the axial reaction force and displacement, were extracted at the RP. Given the regular shape of both the radius and the tibia cross-sections, intensive (material) properties were also computed. Reaction force (N) normalized by mean area (mm^2^) led to stress *σ* (Nmm^−2^), and displacement (mm) divided by initial length (mm) led to strain $\mathrm{\varepsilon}\ \left(-\right)$. These intensive output variables were determined to remove the dependency on the dimensions and shape of the imaged bone sections, consistent with the physical definition where intensive properties are independent of system size.

### Statistical analysis

The study population was stratified by anatomical site (radius, tibia), sex (male, female), and age group (*G*_≤37_: age ≤ 37 yr, *G* _> 37_: age > 37 yr). Given the absence of participants within the 38-40 yr age range in the dataset of young adults, it was decided to lower the threshold to 37 yr to avoid the creation of artificial dependencies on the data. In the younger adults group (*G*_≤37_), a zero-derivative constraint was applied based on the assumption that both densitometric properties (eg, BMC) and geometrical properties (eg, bone cross-sectional area [CSA]) plateau around the age of 18 yr, before progressively declining after the age of 40 yr.^26^ For the older adults group (*G* _> 37_), a constrained quadratic regression of the form *y*(*x*) = *a* + *b* · *x* + *c* · *x*^2^ was fitted to the data. The conditions set out in eqn 2 ensure the continuity between the 2 groups. Baseline values for *G*_≤37_ were estimated by fitting the regression model to the raw data from Stuck et al.^17^ The predicted values from the regression curve were used for subsequent computations. Extensive and intensive values were estimated. The short-term measurement precision error (PE_st_) of densitometric properties was reported within the range of 0*.*6%-1*.*7% for single-stack HRpQCT images by Chiba et al.,^14^ providing more sensitivity than DXA (0*.*77%-1*.*7%^27^). Moreover, the PE_st_ for yield force (F_y_) was reported in the range of 1*.*57%-3*.*29%, indicating that the estimated mechanical behavior does not introduce additional variability.^8^ The coefficient of variation $\left(\frac{\mathrm{\sigma}}{\mathrm{\mu}}\right)$, the PE_st_, the median change per annum $\left(\frac{\Delta \overset{\sim }{\mathrm{\mu}}}{\overset{\sim }{\mathrm{\mu}}}/y\ \left(\%\right)\right)$, and the short-term trend assessment interval (TAI_st_)^28^ were calculated for each property. The latter facilitated the detection of changes that exceeded the PE_st_, which might have otherwise been obscured by statistical noise. Six key size-independent variables were selected based on their clinical relevance for follow-up measurements. Five of these variables exhibited the shortest TAI_st_ (total volumetric BMD (Tot.vBMD), Ct.vBMD, trabecular bone volume over total volume (Tb.BV/TV), relative cortical thickness (Rel.Ct.Th), and apparent yield stress (^app^*σ*_y_)). Lastly, the trabecular degree of anisotropy (Tb.DA) was included due to its independence from the other parameters and its comprehensive characterization of bone microstructural orientation. As reported by Maquer et al.^29^, fabric anisotropy contributes up to 10% to trabecular bone stiffness estimation. The 3 densitometric properties (Tot.vBMD, Tb.BV/TV, and Ct.vBMD) were extracted from the standard evaluation of the scanner’s software. Rel.Ct.Th was calculated as Ct.Th /$\sqrt{\mathrm{Tot}.\mathrm{Ar}/\pi }$, where Ct.Th was evaluated by the scanner’s software as the mean spacing of the periosteal and endocortical surfaces via the distance transformation method, and Tot.Ar represented the mean cross-sectional area of all slices.^30^ The bone diameter was estimated by assuming a mean cylinder geometry, derived from Tot.Ar as $D=2\sqrt{\mathrm{Tot}.\mathrm{Ar}/\pi }$. Tb.DA was estimated as the ratio between the largest and the smallest eigenvectors *m*_3_/*m*_1_ using MSL (MSL^XCTII^),^8^ and was corrected by a power function to the more common mean intercept length (MIL^XCTII^) (see Appendices G and H in the Supplementary material). The ^app^*σ*_y_ was calculated as the yield force divided by the mean total area: ${F}_y/\overline{\mathrm{Tot}.\mathrm{Ar}}$(Nmm^−2^). *Z*-score curves were calculated for all properties, and a novel *radar plot* was elaborated to give a complementary overview of distal bone health based on these 6 parameters. The T-score was computed to evaluate a person’s absolute value relative to the corresponding baseline. T-scores were defined as the value of the participant minus the mean value of the reference population divided by the standard deviation of the reference population (see eqn 3). This definition was first proposed by Dr T. Kelly and was consolidated by the WHO Study Group report in 1994.^31^ Similarly to the well-known T-score used for aBMD, intervals in the range [−3*.*5*,* 2](SD) are provided. The quadratic fit parameters for both healthy female and male cohorts are provided in Tables D6 and D7 in the Supplementary material. Tables 1 and 2 report the descriptive statistics for female and male cohorts for the properties with the biggest yearly variation estimated by TAI_st_, along with FN aBMD and the minimum set of variables to report when describing bone structure, as recommended by Whittier et al.^32^ These properties are force at failure (*F_max_*), cortical thickness (Ct.Th), cortical porosity (Ct.Po), trabecular volumetric BMD (Tb.vBMD), trabecular number (Tb.N), trabecular separation (Tb.Sp), and trabecular thickness (Tb.Th). Data treatment and visualization were performed in Python 3.10 and statistical analysis was performed in R 4.2.0 (2022-2104-22). 


(1) \begin{equation*} y(x)=\left\{\begin{array}{cc}\overline{y_{x\le 37}}& if\;x\le 37\\{}a+b\cdot x+c\cdot{x}^2& if\;x > 37\end{array}\right. \end{equation*}

(2) \begin{equation*} {\displaystyle \begin{array}{c}{\left.\frac{dy}{dx}\right|}_{x=37}=b+2\cdot c\cdot 37=0\\{}y(37)=a+b\cdot 37+c\cdot{37}^2=\overline{y_{x\le 37}}\end{array}} \end{equation*}

(3) \begin{equation*} \mathrm{T}-\mathrm{score}=\frac{X-\mu }{\sigma } \end{equation*}

**Table 1:** Descriptive statistics for the female cohort.

	$\boldsymbol{\mu}$	$\boldsymbol{\sigma}$	$\frac{\boldsymbol{\sigma}}{\boldsymbol{\mu}}$	**PE**_**st**_ **(%)**	$\frac{\boldsymbol{\Delta }\overset{\sim }{\boldsymbol{\mu}}}{\overset{\sim }{\boldsymbol{\mu}}}/\boldsymbol{y}\ \left(\%\right)$	**TAI**_**st**_ **(years)**
**FN BMD (g/cm** ^**2**^ **)**	0.849	0.121	0.143	^c^1.200	−0.603	3.582
**Tibia**						
^**a**^**Tot.vBMD** $\left[\frac{\mathbf{gHA}}{{\mathbf{cm}}^{\mathbf{3}}}\right]$	0.266	0.036	0.135	0.427	−0.753	1.021
^**a**^**Ct.vBMD** $\left[\frac{\mathbf{gHA}}{{\mathbf{cm}}^{\mathbf{3}}}\right]$	0.865	0.032	0.037	0.37	−0.657	1.013
^**a**^**Tb.BV/TV [−]**	0.286	0.044	0.153	0.654	−0.367	3.207
^**a**^**Rel.Ct.Th [−]**	0.065	0.012	0.179	1.315	−1.024	2.312
^**a**^^**app**^***σ***_**y**_$\left[\mathbf{MPa}\right]$	10.988	2.279	0.207	3.514	−1.368	4.625
^**a**^**Tb.DA [−]**	1.726	0.071	0.041	1.582	−0.192	14.822
^**b**^${\mathbf{F}}_{\mathbf{y}}$**[kN]**	9.967	2.187	0.219	4.13	−0.872	8.521
^**b**^**Ct.Th [mm]**	1.096	0.159	0.145	1.388	−0.874	2.86
^**b**^**Ct.Po [−]**	0.015	0.006	0.427	5.176	1.142	8.158
^**b**^**Tb.N [1/mm]**	1.59	0.155	0.098	2.027	0.197	18.518
^**b**^**Tb.Sp [mm]**	0.591	0.069	0.116	1.561	−0.078	36.215
^**b**^**Tb.Th [mm]**	0.251	0.018	0.073	0.605	−0.036	30.175
^**b**^**Tb.vBMD** $\left[\frac{\mathbf{gHA}}{{\mathbf{cm}}^{\mathbf{3}}}\right]$	0.197	0.031	0.158	0.509	−0.41	2.235
**Radius**						
^**a**^**Tot.vBMD** $\left[\frac{\mathbf{gHA}}{{\mathbf{cm}}^{\mathbf{3}}}\right]$	0.297	0.048	0.162	0.591	−1.178	0.903
^**a**^**Ct.vBMD** $\left[\frac{\mathbf{gHA}}{{\mathbf{cm}}^{\mathbf{3}}}\right]$	0.926	0.04	0.043	1.247	−0.414	5.424
^**a**^**Tb.BV/TV [−]**	0.209	0.045	0.215	3.7	−0.479	13.916
^**a**^**Rel.Ct.Th [−]**	0.113	0.021	0.19	3.21	−0.91	6.347
^**a**^^**app**^***σ***_**y**_$\left[\mathbf{MPa}\right]$	13.656	3.959	0.29	5.71	−1.32	7.788
^**a**^**Tb.DA [−]**	1.822	0.103	0.056	1.567	−0.046	61.731
^**b**^${\mathbf{F}}_{\mathbf{y}}$**[kN]**	3.809	0.947	0.249	4.62	−1.718	4.841
^**b**^**Ct.Th [mm]**	1.057	0.154	0.146	1.843	−0.785	4.227
^**b**^**Ct.Po [−]**	0.003	0.002	0.621	11.262	3.047	6.654
^**b**^**Tb.N [1/mm]**	0.145	0.031	0.213	3.314	−0.573	10.406
^**b**^**Tb.Sp [mm]**	1.456	0.177	0.121	2.799	−0.533	9.46
^**b**^**Tb.Th [mm]**	0.655	0.094	0.143	2.866	0.576	8.958
^**b**^**Tb.vBMD** $\left[\frac{\mathbf{gHA}}{{\mathbf{cm}}^{\mathbf{3}}}\right]$	0.215	0.012	0.054	1.96	−0.001	2468.193

Abbreviations: μ: population mean, $\mathrm{\sigma}$: SD, $\frac{\mathrm{\sigma}}{\mathrm{\mu}}$: coefficient of variation, PEst [%]: short-term measurement precision, $\frac{\Delta \overset{\sim }{\mathrm{\mu}}}{\overset{\sim }{\mathrm{\mu}}}/\mathrm{y}\ \left[\%\right]$: percent median change per annum, TAI_st_: short-term trend assessment interval.

a Parameters proposed as reference standard for multi-stack HRpQCT.

b Minimal set of parameters proposed by Whittier et al.^32^

c PE_st_ for FN aBMD by DXA reported by Gugler et al.^19^

**Table 2:** Descriptive statistics for the male cohort.

	$\boldsymbol{\mu}$	$\boldsymbol{\sigma}$	$\frac{\boldsymbol{\sigma}}{\boldsymbol{\mu}}$	**PE**_**st**_ **(%)**	$\frac{\boldsymbol{\Delta }\overset{\sim }{\boldsymbol{\mu}}}{\overset{\sim }{\boldsymbol{\mu}}}/\boldsymbol{y}\ \left(\%\right)$	**TAI** **(years)**
**FN BMD (g/cm** ^**2**^ **)**	0.95	0.164	0.173	^c^1.200	−0.418	5.163
**Tibia**						
^**a**^**Tot.vBMD** $\left[\frac{\mathbf{gHA}}{{\mathbf{cm}}^{\mathbf{3}}}\right]$	0.3	0.047	0.157	0.427	−0.591	1.301
^**a**^**Ct.vBMD** $\left[\frac{\mathbf{gHA}}{{\mathbf{cm}}^{\mathbf{3}}}\right]$	0.813	0.038	0.047	0.37	−0.591	1.126
^**a**^**Tb.BV/TV [−]**	0.347	0.05	0.143	0.654	−0.299	3.937
^**a**^**Rel.Ct.Th [−]**	0.07	0.021	0.296	1.315	−1.44	1.644
^**a**^^**app**^***σ***_**y**_$\left[\mathbf{MPa}\right]$	12.956	3.184	0.246	3.514	−1.408	4.493
^**a**^**Tb.DA [−]**	1.687	0.083	0.049	1.582	−0.066	43.424
^**b**^${\mathbf{F}}_{\mathbf{y}}$**[kN]**	14.775	3.657	0.247	4.13	−1.055	7.045
^**b**^**Ct.Th [mm]**	1.314	0.323	0.246	1.388	−1.274	1.96
^**b**^**Ct.Po [−]**	0.028	0.01	0.379	5.176	0.393	23.703
^**b**^**Tb.N [1/mm]**	1.704	0.177	0.104	2.027	−0.067	54.715
^**b**^**Tb.Sp [mm]**	0.545	0.068	0.124	1.561	0.318	8.843
^**b**^**Tb.Th [mm]**	0.275	0.024	0.089	0.605	−0.047	23.165
^**b**^**Tb.vBMD** $\left[\frac{\mathbf{gHA}}{{\mathbf{cm}}^{\mathbf{3}}}\right]$	0.238	0.036	0.151	0.509	−0.342	2.676
**Radius**						
^**a**^**Tot.vBMD** $\left[\frac{\mathbf{gHA}}{{\mathbf{cm}}^{\mathbf{3}}}\right]$	0.335	0.051	0.153	0.591	−0.585	1.82
^**a**^**Ct.vBMD** $\left[\frac{\mathbf{gHA}}{{\mathbf{cm}}^{\mathbf{3}}}\right]$	0.887	0.035	0.04	1.247	−0.229	9.798
^**a**^**Tb.BV/TV [−]**	0.291	0.05	0.172	3.7	−0.487	13.68
^**a**^**Rel.Ct.Th [−]**	0.115	0.025	0.214	3.21	−0.62	9.32
^**a**^^**app**^***σ***_**y**_$\left[\mathbf{MPa}\right]$	16.424	4.285	0.261	5.71	−1.126	9.128
^**a**^**Tb.DA [−]**	1.803	0.108	0.06	1.567	−0.087	32.587
^**a**^${\mathbf{F}}_{\mathbf{y}}$**[kN]**	6.015	1.669	0.277	4.62	−0.328	25.375
^**a**^**Ct.Th [mm]**	1.226	0.207	0.169	1.843	−0.588	5.643
^**a**^**Ct.Po [−]**	0.007	0.006	0.776	11.262	2.367	8.564
^**a**^**Tb.N [1/mm]**	0.199	0.034	0.17	3.314	−0.548	10.885
^**a**^**Tb.Sp [mm]**	1.583	0.234	0.148	2.799	−0.442	11.394
^**a**^**Tb.Th [mm]**	0.568	0.072	0.127	2.866	0.657	7.854
^**a**^**Tb.vBMD** $\left[\frac{\mathbf{gHA}}{{\mathbf{cm}}^{\mathbf{3}}}\right]$	0.242	0.017	0.069	1.96	0.083	42.676

Abbreviations: μ: population mean, $\mathrm{\sigma}$: SD, $\frac{\mathrm{\sigma}}{\mathrm{\mu}}$: coefficient of variation, PEst [%]: short-term measurement precision, $\frac{\Delta \overset{\sim }{\mathrm{\mu}}}{\overset{\sim }{\mathrm{\mu}}}/\mathrm{y}\ \left[\%\right]$: percent median change per annum, TAI_st_: short-term trend assessment interval.

a Parameters proposed as reference standard for multi-stack HRpQCT.

b Minimal set of parameters proposed by Whittier et al.^32^

c PE_st_ for FN aBMD by DXA reported by Gugler et al.^19^

## Results

### Descriptive statistics

**Figure 3:**
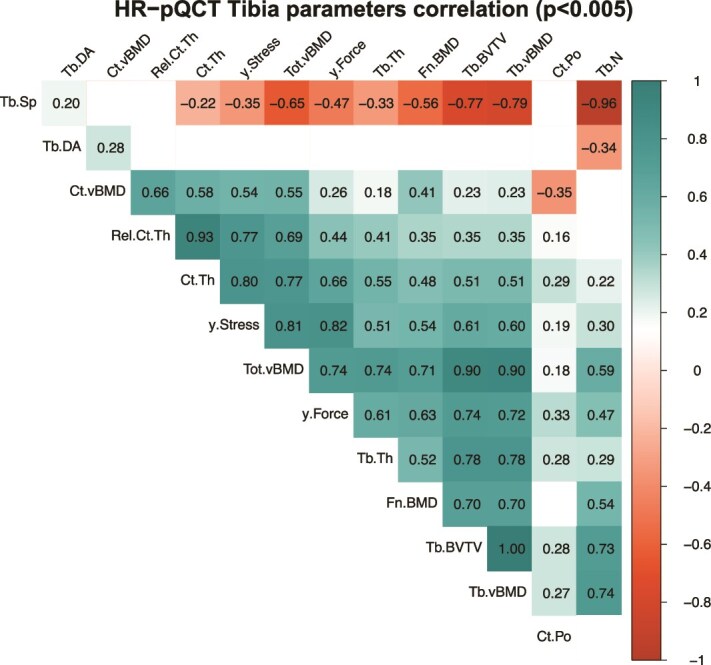
Pearson’s correlation coefficients for standard HRpQCT parameters according to the scanner manufacturer at the tibia for a healthy young population (*n* = 114). Most of the properties derived from Tb.vBMD show correlations *r* ≥ 0.72 (Tb.BV/TV, Tb.N, Tb.Sp, Tb.Th). Non-significant correlations (*p* > .005) were hidden from the plots. Abbreviations: Tb.vBMD, trabecular volumetric BMD; Tb.BV/TV, trabecular bone volume over total volume; Tb.N, trabecular number; Tb.Sp, trabecular separation; Tb.Th, trabecular thickness.

A multiple regression analysis was conducted to evaluate the associations between cortical and trabecular parameters proposed by the scanner manufacturer. Figure 3 shows the 2-tailed Pearson’s correlation test matrix summarizing the output correlation coefficients *r* for cortical and trabecular parameters estimated at the tibia for the healthy young reference *G*_≤37_. Almost all parameters had a strong, significant correlation, except for correlations between geometric and densitometric values. The best predictors of estimated S and F_y_ were Tot.vBMD and Tb.BV/TV. Most of the properties derived from Tb.vBMD show correlations *r* ≥ 0*.*72 (trabecular bone volume over total volume [Tb.BV/TV], Tb.N, Tb.Sp, Tb.Th). Ct.Po shows the overall weakest correlations. There is a weak but significant negative relation between Tb.DA and Tb.N. The non-significant correlations (*p* > *.*005) were hidden from the plots. Results for the radius are provided in Appendix E and Figure E9 in the Supplementary material.

Tables 1 and 2 show the descriptive statistics for the female and male cohorts, respectively. The tables present a summary of the PE_st_, the median change per annum, and the TAI_st_ for a set of newly proposed properties. Additionally, they present the minimum set of parameters to be reported as proposed by Whittier et al.^32^ All properties demonstrated negative trends except for Ct.Po and Tb.Sp, exhibiting analogous patterns for the radius and tibia across both female and male subjects. FN aBMD and Tot.vBMD at the tibia showed similar $\frac{\Delta \overset{\sim }{\mu }}{\overset{\sim }{\mu }}$/y (%) in females (−0*.*603% and −0*.*753%, respectively), and in males (−0*.*418% and −0*.*591%, respectively). Furthermore, tibia Tot.vBMD showed a shorter TAI_st_ with respect to FN aBMD (1*.*02 and 3*.*58 yr for females, and 1*.*30 and 5*.*16 yr for males, respectively). Among the female cohort, Ct.vBMD exhibited the shortest TAI_st_ at the tibia (1*.*01 yr), while Tot.vBMD demonstrated the minimum TAI_st_ at the radius (0*.*90 yr). The same trends were reported for males (1*.*12 and 1*.*82 yr for Ct.vBMD and Tot.vBMD, respectively). The TAI_st_ was substantially smaller for Ct.vBMD than for Ct.Po (1*.*013% and 8*.*158%, respectively, at the female tibia). The same trend is consistent across both sites and sexes, with the exception of the male radius, which is attributable to smaller changes per annum in Ct.Po. Nevertheless, Ct.vBMD, which reflects porosities at all scales, showed a markedly lower *PE_st_* (1*.*247%) compared with Ct.Po (11*.*262%), the latter being dependent on scanner resolution. In both sexes, the geometrical property Rel.Ct.Th showed a shorter TAI_t_ at the tibia with respect to the size-dependent Ct.Th, but the trends were inverted at the radius. The intensive mechanical variable ^app^*σ*_y_ exhibited a shorter TAI_st_ compared with the extensive mechanical property F_y_ across both sites and gender groups, with the exception of the female radius (7*.*79 vs 4*.*84 yr).

### Radar plots

**Figure 4:**
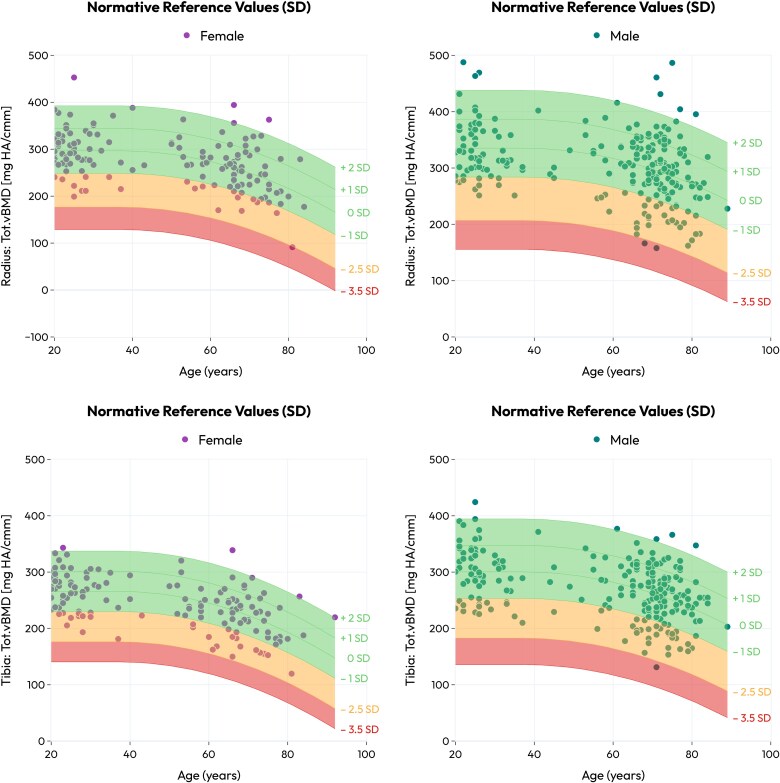
Proposed *Z*-score curves for males and females trabecular volumetric BMD at the radius (a, b), and at the tibia (c, d). The green interval denotes values in the range [−1, 2](SD), the yellow interval expresses the range [−2.5, −1](SD), and the red interval denotes values within the range [−3.5, −2.5](SD). The average value in the 18-37 yr is described as a constant value, while a constrained quadratic fit describes the evolution of the property with age in the interval 37-92 yr. The quadratic fit parameters for both healthy female and male cohorts are provided in Tables D6 and D7 in the Supplementary material.

**Figure 5:**
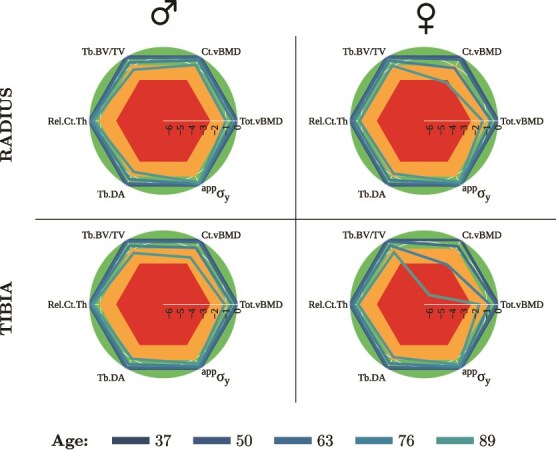
Radar plots exhibiting the relative change from baseline for 4 evenly spaced age subgroups. Cortical volumetric BMD was found to be the property most sensitive to age in the load-bearing tibia of females. The trends were qualitatively similar for both sites and sexes, but their magnitude varied greatly.

Figure 4 presents a representative normative curve for Tot.vBMD at the radius (4a, 4b), and at the tibia (4c, 4d). Given the difficulty of representing 6 properties in 2 dimensions, a *radar plot* was designed to visualize the combined trends of the 6 properties with the most significant yearly change in the form of T-scores (Figure 5). The change over age was calculated from the baseline (37 yr) for 4 evenly spaced age subgroups (50, 63, 76, and 89 yr). These include 2 densitometric properties (Tot.vBMD, Ct.vBMD), 1 size-independent geometric property (Rel.Ct.Th), 2 microstructural properties (Tb.DA, Tb.BV/TV), and 1 intensive mechanical property (^app^*σ*_y_). The trends were more evident in the tibia than in the radius, except for Tb.BV/TV. Interestingly, the latter exhibited a comparable quantitative decrease at both sites. Ct.vBMD at the distal tibia was found to be the property that decreases most with age, especially between the sixth and eighth decades of life in females. It was followed by Tot.vBMD, Tb.BV/TV, Tb.DA, Rel.Ct.Th, and finally by ^app^*σ*_y_.

### hFE to assess mechanical behavior

The FE analyses were performed using a novel pipeline.^8^ The computational time was 16*.*7 min (14*.*0-21*.*7 min) for triple-stack tibia images and 13*.*8 min (12*.*1-16*.*8 min) for double-stack radius images (median, [IQR]), respectively. A comparison with the previously presented method by Arias-Moreno et al.^5^ is provided in Appendix F in the Supplementary material.

## Discussion

This study provides the first age-, sex-, and site-specific normative data for multi-stack second-generation HRpQCT of the distal skeleton. The collected data included a total of 381 healthy participants (144 F, 237 M) from a primarily Caucasian population, aged 20-92 yr. The derived *Z*-score curves are a valuable tool for comparing an individual or a population of interest to a reference cohort without recruiting a control group in cross-sectional studies.

The interest in developing these multi-stack reference data is 2-fold. Firstly, as pointed out by Warden et al.^13^, it remains unclear whether ultra-distal or diaphyseal ROIs alone correspond with the location where fractures occur. The use of multi-stack protocols was promoted by the findings of Baumbach et al.^14^, who highlighted the distribution of Colles’ fractures at the radius. However, experimental testing on ex vivo human distal tibiae has revealed no precise physiological fracture location.^33^ Multi-stack radius images facilitate a detailed assessment of the region susceptible to Colles’ fractures,^14^ while the tibia functions as a surrogate for FN strength, presumably attributable to the weight-bearing function of the limb.^34^ The use of T-scores calculated from a healthy and young reference is justified by the perspective that osteoporosis originates in early life but manifests its consequences in old age,^35^ and not only the relative amount of bone lost but also the total amount of bone gained in early adulthood is indicative of bone strength in elderly individuals who are susceptible to fractures.^26^ Secondly, multi-stack protocols offer several advantages due to their bigger ROI, such as a bigger LCH in longitudinal studies, albeit with limited disadvantages, including a minor increase in radiation dose, stack shifts,^32^ or scanning time.^15^ The double-stack protocol doubles scanning time from ~2 to 4 min, while the triple-stack extends it to ~6 min. The hFE analysis was not significantly affected by the increased stack thickness. The increased risk of motion artifacts due to the longer scanning time did not substantially influence multi-stack images, especially at the distal tibia, as supported by findings from the AFFIRM-CT cohort: 21% of radius scans and 4% of tibia scans necessitated a secondary acquisition due to motion artifacts exceeding a score of 3/5 on the scale proposed by Pialat et al.^21^ These rates are consistent with those previously reported by Pialat et al.^21^ for single-stack images (radius: 29*.*7%, tibia: 15*.*7%).

Previous works calculated centile curves based on generalized additive models for location, scale, and shape (GAMLSS) for first-generation in a Caucasian population^11^ and in a Chinese population,^36^ and for second-generation HRpQCT in Caucasian populations.^12^^,^^13^ This technique can accommodate high skew or kurtosis distributions. However, its high complexity can potentially influence the interpretation of some parameters. For instance, several reversals of the trend with age have been observed for Tb.vBMD, Tb.BV/TV, and Tb.Th,^13^ although these findings were not consistent with those of Warming et al.^37^, who found a constant decrease in aBMD at the hip and distal forearm. Moreover, in the distribution’s tails, LMS curves are sensitive to individuals.^13^ Consequently, a quadratic regression was identified as the optimal compromise between simplicity and robustness, particularly in light of the limited presented cohort.

Six size-independent variables with the highest sensitivity to age were selected for further analysis. These include 2 densitometric properties (Tot.vBMD, Ct.vBMD), 1 size-independent geometric property (Rel.Ct.Th), 2 microstructural properties (Tb.DA, Tb.BV/TV), and 1 intensive mechanical property (^app^*σ*_y_). Density measurements were chosen because they are less sensitive to partial volume effects and motion artifacts,^12^ and they were the strongest predictors of estimated apparent yield stress (^app^*σ*_y_) as displayed in Figure B6 in the Supplementary material. These variables were then visualized on a radar plot to improve the understanding of an individual’s results with respect to a healthy young reference (Figure 5). Ct.vBMD showed the highest sensitivity with age. Our findings agree with Warden et al.^13^, who observed a more pronounced decline in density in females at both the distal and diaphyseal sites.

The choice of Ct.vBMD over Ct.Po was motivated by the shorter TAI_st_ in Ct.vBMD as described in Tables 1 and 2, suggesting that Ct.vBMD is a more robust descriptor of cortical structural density at the HRpQCT resolution. This can be attributed to the ability of Ct.vBMD to capture porosities at all scales via X-ray attenuation, whilst Ct.Po cannot detect pores smaller than its resolution at 10% MTF (∼92*.*5 to 112*.*6 μm), although over 60% of human cortical pores are smaller than 100 μm in diameter.^38^ These findings were consistent with existing literature by Whittier et al.^32^ who reported low precision in Ct.Po. In addition, using whole bone densitometric parameters provides more robust outcomes for assessing bone health at distal skeletal sites.^13^ The high correlation between the latter and the derived microstructural parameters suggests partial redundancy in reporting them all in predicting individual fracture risk, as the latter is mostly influenced by structural density (Figure 3 and Figure E9 in the Supplementary material) and anisotropy.^29^ The present study further supports other findings that describe how trabecular and cortical densities behave differently with age.^4^^,^^36^ It is, therefore, beneficial to report both. It is hypothesized that periosteal expansion serves a protective role by increasing the CSA, thereby increasing the moment of inertia. Consequently, this results in a reduction of stress and an increase in the capacity of the bone to store strain energy. The investigation revealed that size-independent geometric properties are more sensitive to short-term variability. A representation of cortical thickness relative to the diameter of the bone was proposed (Rel.Ct.Th). This normalization facilitates the consideration of initial bone size, thereby enabling the evaluation of thinning relative to the initial size. In contrast, Ct.Th demonstrates variations relative to its value at baseline. Moreover, size-independent geometric properties helped with the elimination of implausible global maxima, such as the peak observed at 48 yr of age in females for Ct.Th reported by Zhu et al.^36^ This phenomenon is presumably attributable to the segmentation of HRpQCT images, which is based on density thresholds and is affected by interoperator variability,^39^ resulting in an overestimation of cortical bone changes.^40^ Tb.DA showed a negative trend with age at both distal locations. This was consistent with findings by Doi et al.,^41^ but showed different trends from what was reported at the lumbar spine by Mosekilde et al.^42^ Tb.DA reflects the microstructural anisotropy in the trabecular compartment and is uncoupled from Tb.BV/TV^43^ (further supported by Figure 3 and Figure E9 in the Supplementary material). As demonstrated in previous studies, the fabric tensor is the second most significant contributor to trabecular bone stiffness.^29^ Additionally, the latter is an easily accessible parameter, as it is readily available with the MIL estimation of the scanner manufacturer. The present study provides an estimation based on MSL, which is then converted to MIL to facilitate comparability (see Appendix G in the Supplementary material). Finally, the intensive mechanical property ^app^*σ*_y_ describes the mechanical behavior of the bone section with a smaller PE_st._^8^ While failure load showed the most significant association with fracture risk,^44^ it is dependent on the bone size. Hence, ^app^*σ*_y_ better reflects bone quality at the material level and may be more meaningful for longitudinal bone monitoring.

Multi-stack densitometric properties showed good agreement with their single-stack counterparts, indicating the potential for cross-study and cross-protocol comparisons (see Appendix C in the Supplementary material). In contrast, size-independent geometrical parameters describe the predominance of cortical thickness in the single-stack sections, as it thins towards the distal end. These findings are consistent with those reported by Boyd.^45^ The multi-stack models exhibit lower S and lower correlations in the radius and tibia, which can be attributed to St-Venant’s effect associated with the highly constrained boundary conditions in flat single sections. In contrast, multi-stack models exhibit a greater F_y_ due to their larger volumetric domain that allows for greater strain energy storage under equivalent boundary conditions.

The novel pipeline showed a good correlation for F_y_ against the version provided by the scanner manufacturer and published by Arias-Moreno et al.^5^ Conversely, the novel pipeline consistently yielded higher S relative to the previous implementation. This was likely due to the improved representation of the cortical geometry with respect to the previous method, which used thick isotropic elements of ∼1*.*7 mm edge length and mixed-phase elements. Furthermore, the FE analysis pipeline used in this study^8^ was 90% faster than its predecessor.^6^ The time difference between the radius and tibia was not significant, although the radius images are limited to 2 consecutive stacks in thickness with respect to the 3 tibia images. The difference was not significant because the degrees of freedom of the mesh were the same, and the moderate time gain in radius analysis is attributed to the shorter time spent on creating the segmented surface.

## Limitations

Despite the limited sample size in comparison with analogous normative data,^11–13^^,^^36^ the present study demonstrated comparable trends with the previously reported literature. Additionally, the study population was predominantly of Caucasian background, which may limit the generalizability of the findings to other racial and ethnic populations. The methodology employed in this study is subject to certain limitations, including the pragmatic definition of the age threshold at 37 yr, despite similar trends having been shown in previous studies.^12^^,^^13^ Furthermore, the quadratic fit employed in this study is unable to capture the accelerated bone loss that occurs around menopause, although it is beneficial in reducing artificial reversals in trends typically observed in GAMLSS analyses.

## Conclusion

This work established the first age-, sex-, and site-specific reference data for multi-stack second-generation HRpQCT of the distal radius and tibia. Six size-independent parameters demonstrated superior sensitivity to age-related skeletal changes compared with the recommended set of properties to report for single stacks. Ct.vBMD at the weight-bearing tibia of women was the most sensitive to age, particularly between the sixth and eighth decades of life. Multi-stack protocols have demonstrated strong agreement with single-stack densitometric measurements, thus allowing for cross-study comparisons while addressing the limitations imposed by the thin single-stack sections. Using intensive properties eliminated the confounding effects of skeletal dimensions, thereby providing a more sensitive detection of material-level bone changes that may be obscured by dimensional effects. The proposed *radar plots* offer a more comprehensive overview of bone health beyond standard densitometric and microstructural parameters. These findings further show the potential of HRpQCT, despite its limited worldwide availability, as a valuable diagnostic tool beyond conventional FN aBMD by DXA, especially in ambiguous fracture risk stratification, monitoring treatment response, and assessing rapid changes (eg, entering menopause or chronic glucocorticoid use). Future research should establish new thresholds for clinical interventions that integrate both microstructural and mechanical determinants of bone fragility alongside fall risk assessment and investigate their sensitivity to therapeutic interventions.

## Supplementary Material

reference_data_main-supplementary-revA_ziag077

## Data Availability

The processing code supporting the findings of this study is publicly available.^46^ The underlying data cannot be made publicly available owing to privacy and ethical constraints; however, data may be made available upon reasonable request to the corresponding author.
