# Supplementary material for: Sex- and site-specific reference data for size-invariant properties using multi-stack HRpQCT
Source: JBMR Plus. 2026 Apr 25;10(6):ziag077. doi: 10.1093/jbmrpl/ziag077 (PMC13198809; doi:10.1093/jbmrpl/ziag077)
Supplement: reference_data_main-supplementary-revA_ziag077 [file reference_data_main-supplementary-reva_ziag077.pdf]

Appendix A. Participant demographics

Table A.4: Age (years), Femoral neck aBMD ($g\,cm^{-2}$), BMI ($kg\,m^{-3}$), and FRAX (%) statistics for participants under 40 years.

	Females ($n = 61$)	Males ($n = 68$)	p-value*
Age (years)			
Mean (sd)	25.25 (4.57)	26.24 (4.45)	0.22
Median [lq, uq]	24.00 [21.00, 28.00]	25.00 [22.75, 29.25]	0.13
BMI ($kg\,m^{-3}$)			
Mean (sd)	22.24 (2.66)	23.81 (2.90)	0.002
Median [lq, uq]	22.18 [20.62, 22.99]	23.29 [22.13, 25.51]	$p < 0.001$
Femoral neck aBMD ($g\,cm^{-2}$)			
Mean (sd)	0.85 (0.12)	0.95 (0.16)	$p < 0.001$
Median [lq, uq]	0.84 [0.77, 0.94]	0.96 [0.82, 1.06]	$p < 0.001$
FRAX: MOF (%)			
Mean (sd)	5.26 (2.01)	4.94 (1.90)	0.34
Median [lq, uq]	4.20 [3.45, 7.15]	4.05 [3.30, 6.50]	0.09
FRAX: Hip Fracture (%)			
Mean (sd)	0.37 (0.59)	0.36 (0.67)	0.93
Median [lq, uq]	0.10 [0.00, 0.50]	0.10 [0.00, 0.30]	0.30

*Student's t-test for continuous variables presented with mean (sd) and the Wilcoxon-Mann-Whitney test for continuous variables presented with median [lq, uq]. *sd, standard deviation; lq, lower quartile; uq, upper quartile; aBMD, areal bone mineral density; MOF, major osteoporotic fracture.*

Table A.5: Age (years), Femoral neck aBMD ($g\,cm^{-2}$), BMI ($kg\,m^{-3}$), and FRAX (%) statistics for participants over 40 years.

	Females (n = 83)	Males (n = 169)	p-value*
Age (years)			
Mean (sd)	65.84 (9.66)	70.23 (8.02)	$p < 0.001$
Median [lq, uq]	66.00 [59.50, 72.00]	71.00 [66.00, 75.00]	$p < 0.001$
BMI ($kg\,m^{-3}$)			
Mean (sd)	24.53 (4.11)	27.34 (4.11)	$p < 0.001$
Median [lq, uq]	23.67 [21.64, 26.99]	26.78 [24.51, 29.41]	$p < 0.001$
Femoral neck aBMD ($g\,cm^{-2}$)			
Mean (sd)	0.72 (0.13)	0.79 (0.12)	$p < 0.001$
Median [lq, uq]	0.70 [0.62, 0.79]	0.79 [0.70, 0.87]	$p < 0.001$
FRAX: MOF (%)			
Mean (sd)	13.29 (8.83)	7.80 (4.70)	$p < 0.001$
Median [lq, uq]	11.00 [7.20, 16.00]	6.90 [5.20, 8.80]	$p < 0.001$
FRAX: Hip Fracture (%)			
Mean (sd)	3.35 (5.87)	2.12 (3.53)	0.09
Median [lq, uq]	1.40 [0.35, 3.40]	1.40 [0.50, 2.40]	0.41

*Student's t-test for continuous variables presented with mean (sd) and the Wilcoxon-Mann-Whitney test for continuous variables presented with median [lq, uq]. *sd, standard deviation; lq, lower quartile; uq, upper quartile; aBMD, areal bone mineral density; MOF, major osteoporotic fracture.*

Appendix B. Correlations of extensive and intensive variables

Intensive densitometric variables were stronger predictors of mechanical properties than extensive variables. The predictions were estimated through quadratic regressions. Figures B.6b, B.6d show the relationship between Tot.vBMD and ${}^{\mathrm{app}}\sigma_{\mathrm{y}}$ ($R^2 = 0.59 - 0.69$, $p < 0.001$). There is a clear overlap between the female and male groups for the intensive properties. In contrast, B.6a, B.6c show the extensive densitometric property total bone mineral content (Tot.BMC) with a weaker relationship with $\mathrm{F_y}$ which was statistically significant ($R^2 = 0.48 - 0.59$, $p < 0.001$).

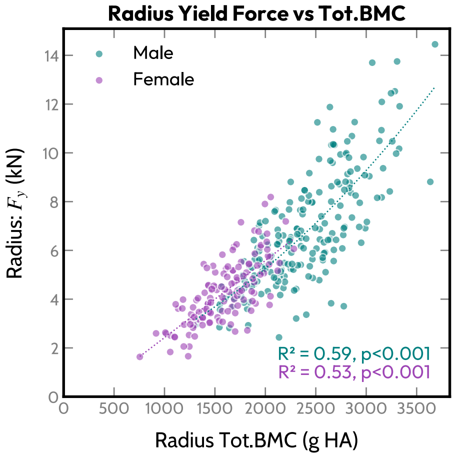

(a) Relationship between F_y and Tot.BMC at the radius.

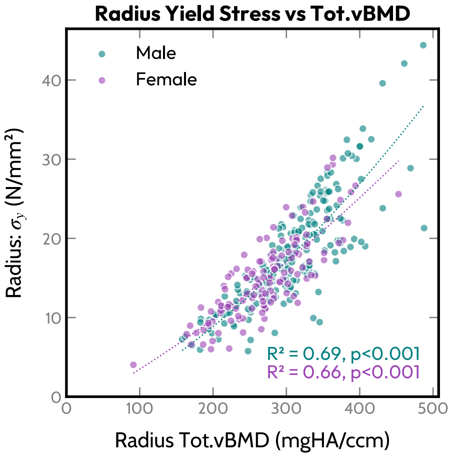

(b) Relationship between ${}^{app}\sigma_y$ and Tot.vBMD at the radius.

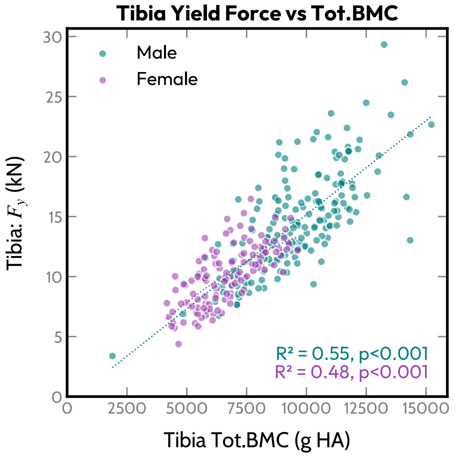

(c) Relationship between F_y and Tot.BMC at the tibia.

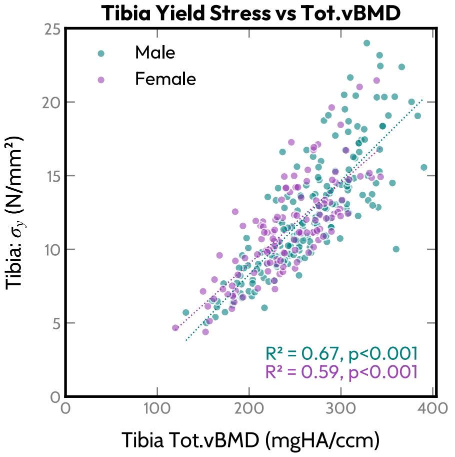

(d) Relationship between ${}^{app}\sigma_y$ and Tot.vBMD at the tibia.

Figure B.6: Different relationships between extensive and intensive variables. The intensive variables show better agreement and statistical significance with each other.

Appendix C. Multi-stack to single-stack Comparison

Second-generation HR-pQCT images were acquired from healthy, young participants (22 years to 37 years, M=45, F=44) in previous studies at our clinic using a multi-stack protocol. The images were cropped to their single-section equivalents using the relative offset method to adjust for forearm length. If not fully covered, cropping occurred at the most distal stack. Missing forearm lengths were estimated using an anthropometric relation based on the participant's height. Image processing followed the standard workflow from the scanner's manufacturer (IPL Scanco Module 64-bit, Version V5.16).

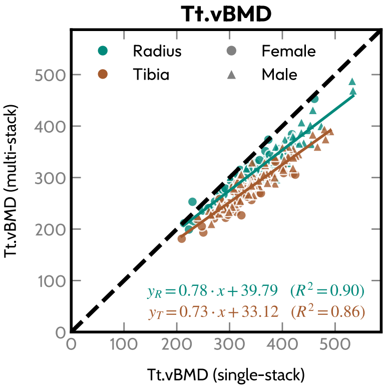

(a) Multi- vs. single-stack Tot.vBMD.

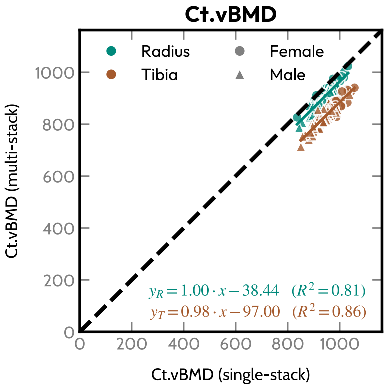

(b) Multi- vs. single-stack Ct.vBMD.

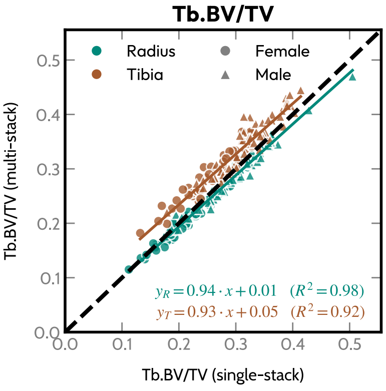

(c) Multi- vs. single-stack Tb.BV/TV.

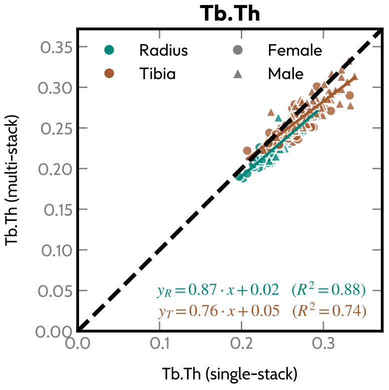

(d) Multi- vs. single-stack Tb.Th.

Figure C.7: Multi- vs. single-stack comparison of densitometric (total volumetric bone mineral density (Tot.vBMD), cortical volumetric bone mineral density (Ct.vBMD)) and microstructural properties (trabecular bone volume over total volume (Tb.BV/TV), trabecular thickness (Tb.Th)).

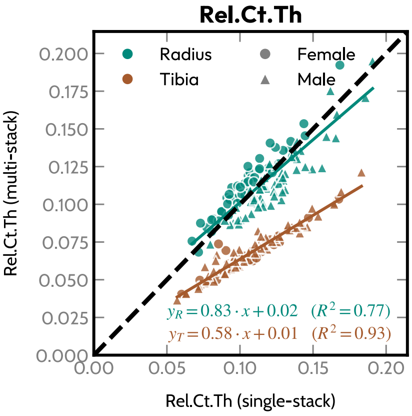

(a) Multi- vs. single-stack Rel.Ct.Th.

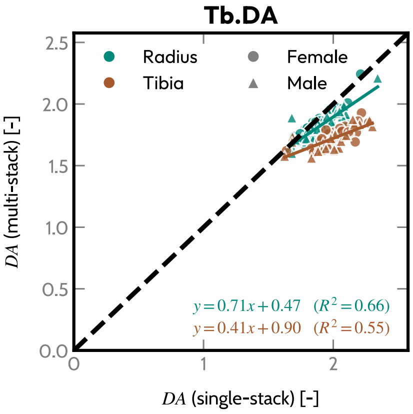

(b) Multi- vs. single-stack Tb.DA.

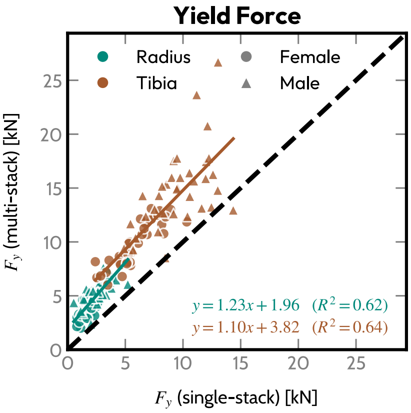

(c) Multi- vs. single-stack F_y.

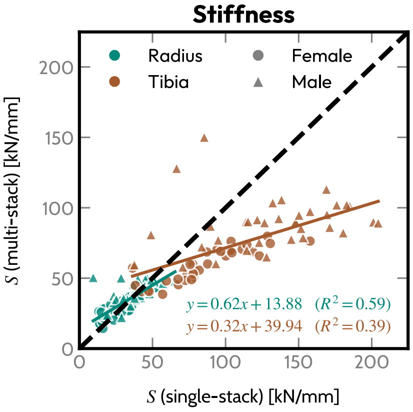

(d) Multi- vs. single-stack S.

Figure C.8: Multi- vs. single-stack comparison of size-independent geometrical (relative cortical thickness (Rel.Ct.Th)), microstructural (trabecular degree of anisotropy (Tb.DA)), and extensive mechanical properties (yield force (F_y), stiffness (S)).

Table D.6: Quadratic fit parameters for the healthy female cohort.

Property	μ_{ref}	σ_{ref}	a	b	c
Tibia					
Tot.vBMD [$\frac{\mathrm{mgHA}}{\mathrm{cm}^3}$]	265.777966	35.776481	212.082551	2.902455	-0.039222
Ct.vBMD [$\frac{\mathrm{mgHA}}{\mathrm{cm}^3}$]	865.106780	32.034264	704.663108	8.672631	-0.117198
Tb.BV/TV [-]	0.286068	0.043741	0.237668	0.002616	-0.000035
Rel.Ct.Th [-]	0.064878	0.011610	0.057115	0.000420	-0.000006
Tb.DA [-]	1.725506	0.071044	1.651882	0.003980	-0.000054
${}^{\mathrm{app}}\sigma_{\mathrm{y}}$ [MPa]	10.988131	2.278751	10.181289	0.043613	-0.000589
Radius					
Tot.vBMD [$\frac{\mathrm{mgHA}}{\mathrm{cm}^3}$]	296.648333	47.944599	237.426327	3.201190	-0.043259
Ct.vBMD [$\frac{\mathrm{mgHA}}{\mathrm{cm}^3}$]	925.761667	39.646741	832.690784	5.030859	-0.067985
Tb.BV/TV [-]	0.208917	0.044961	0.171972	0.001997	-0.000027
Rel.Ct.Th [-]	0.112519	0.021423	0.096137	0.000886	-0.000012
Tb.DA [-]	1.822080	0.102901	1.714980	0.005789	-0.000078
${}^{\mathrm{app}}\sigma_{\mathrm{y}}$ [MPa]	13.656021	3.959029	12.587445	0.057761	-0.000781

The provided function parameters are in the form $y(x) = a + b \cdot x + c \cdot x^2$.

Table D.7: Quadratic fit parameters for the healthy male cohort.

Property	μ_{ref}	σ_{ref}	a	b	c
Tibia					
Tot.vBMD [$\frac{\text{mgHA}}{\text{cm}^3}$]	300.400000	47.018859	252.716539	2.577484	-0.034831
Ct.vBMD [$\frac{\text{mgHA}}{\text{cm}^3}$]	813.336765	38.283101	752.845254	3.269811	-0.044187
Tb.BV/TV [-]	0.346868	0.049557	0.288373	0.003162	-0.000043
Rel.Ct.Th [-]	0.069709	0.020612	0.063386	0.000342	-0.000005
Tb.DA [-]	1.686837	0.082738	1.613005	0.003991	-0.000054
${}^{\text{app}}\sigma_{\text{y}}$ [MPa]	12.955912	3.184268	11.511722	0.078064	-0.001055
Radius					
Tot.vBMD [$\frac{\text{mgHA}}{\text{cm}^3}$]	335.313235	51.357454	288.288420	2.541882	-0.034350
Ct.vBMD [$\frac{\text{mgHA}}{\text{cm}^3}$]	886.783824	35.394669	859.842625	1.456281	-0.019679
Tb.BV/TV [-]	0.291059	0.049987	0.231716	0.003208	-0.000043
Rel.Ct.Th [-]	0.114675	0.024552	0.106112	0.000463	-0.000006
Tb.DA [-]	1.803155	0.107955	1.674065	0.006978	-0.000094
${}^{\text{app}}\sigma_{\text{y}}$ [MPa]	16.423988	4.285244	15.476789	0.051200	-0.000692

The provided function parameters are in the form $y(x) = a + b \cdot x + c \cdot x^2$.

Appendix E. Correlation matrix for HR-pQCT parameters at the distal radius

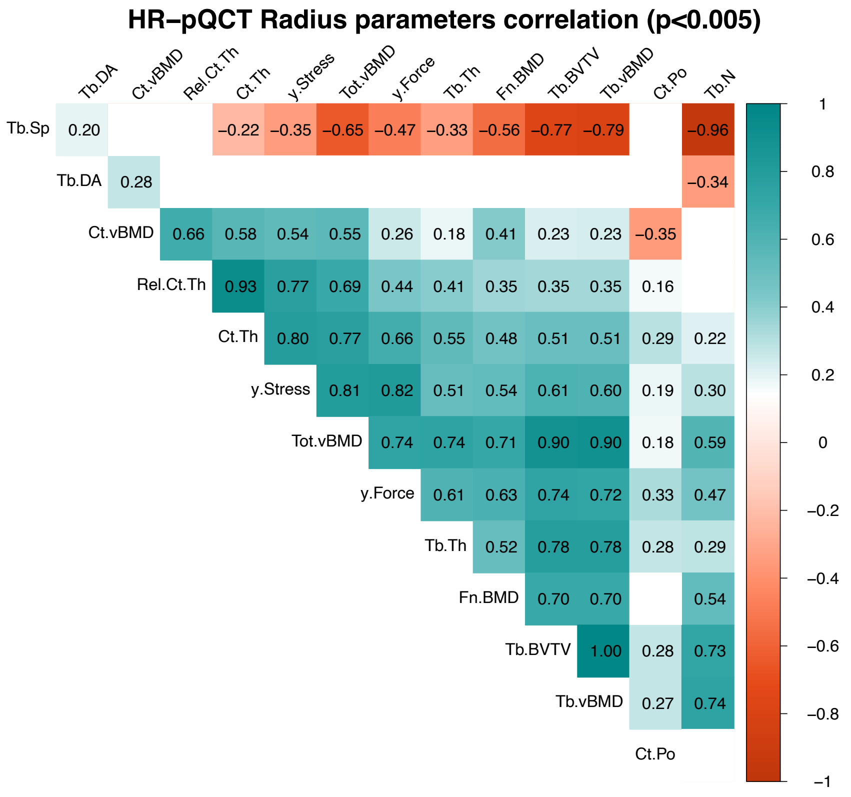

Figure E.9: Pearson's correlation coefficients for standard HR-pQCT parameters according to the scanner manufacturer at the radius for a healthy young population ($n = 114$). Most of the properties derived from Tb.vBMD show correlations $r > .72$ (Tb.BV/TV, Tb.N, Tb.Sp, Tb.Th). Non-significant correlations ($p > 0.005$) were hidden from the plots.

Appendix F. Comparison between Arias-Moreno et al. (2019) and the newly proposed method.

The FE analyses were performed using a novel pipeline. As illustrated in Figure F.10a, there appears to be a robust correlation with the F_y calculated with the method proposed by Arias-Moreno et al. The linear regressions yielded $y = 0.90 \cdot x + 0.2\text{kN}(R^2 = 0.75)$ and $y = 1.14 \cdot x - 0.91\text{kN}(R^2 = 0.81)$ for radius and tibia, respectively. As illustrated in Figure F.10c, S showed an overestimation of the novel method with respect to the method proposed by Arias-Moreno and colleagues of $y = 0.73 \cdot x + 4.91\text{kN}^2\,\text{mm}^{-1}(R^2 = 0.70)$ and $y = 0.79 \cdot x - 6.40\text{kN}^2\,\text{mm}^{-1}(R^2 = 0.81)$ for radius and tibia, respectively. The Bland-Altman plots (Figures F.10b, F.10d) revealed differences between the proposed hFE pipeline and the method proposed by Arias-Moreno et al. For the estimation of mechanical properties, a greater bias was shown in the tibia. For the estimated force, the radius showed a mean difference of 0.314 kN (95 % LoA: −1.849 – 2.477 kN), while the tibia showed a mean difference of −0.845 kN (95 % LoA: −5.355 – 3.664 kN). For the estimated stiffness, the radius showed a mean difference of 5.419 kN mm^{-1} (95 % LoA: −9.819 – 20.658 kN mm^{-1}), while the tibia showed a mean difference of 22.354 kN mm^{-1} (95 % LoA: 3.723 – 40.986 kN mm^{-1}).

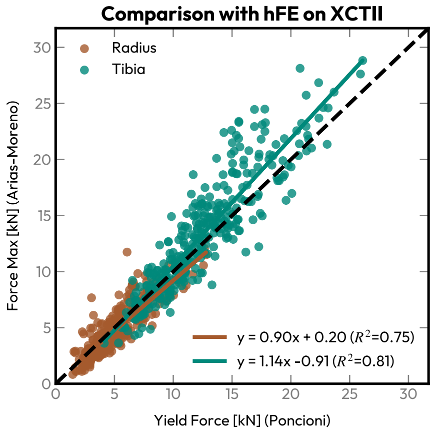

(a) Linear regression on estimated force.

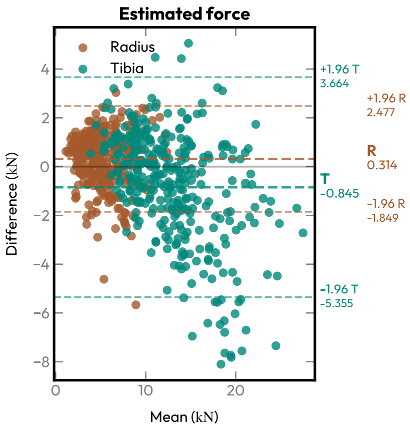

(b) Bland-Altman plot on estimated force.

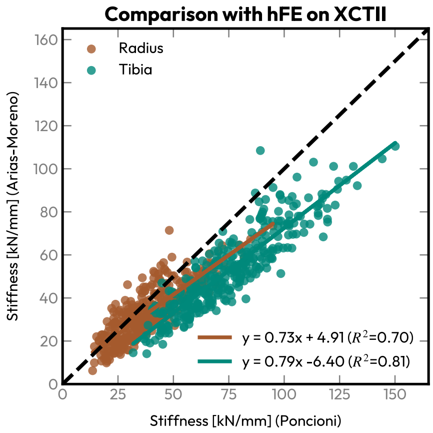

(c) Linear regression on estimated stiffness.

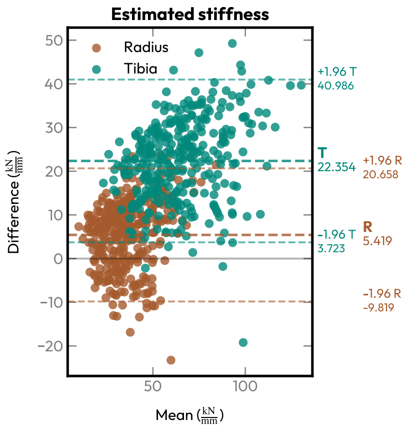

(d) Bland-Altman plot on estimated stiffness.

Figure F.10: Comparison between primary outputs of the recently developed pipeline and the version proposed by Arias-Moreno et al. (2019). A robust correlation with force was observed; however, the new hFE pipeline overestimated stiffness.

Appendix G. Overview of the relationships of structural density (Tb.BVTV) and of the fabric tensor

Figure G.11 shows the calibration coefficients of the fabric anisotropy eigenvalues using the mean surface length (**MSL**) against mean intercept length (**MIL**) on the segmented HR-pQCT images at 61 µm resolution, and with the fabric anisotropy estimated by the gold standard **MIL** on the segmented micro computed tomography (μCT) images at 16.4 µm resolution. As the elastic properties of the constitutive model are derived at the resolution of the μCT scan, a linear combination of the coefficients proposed by Hosseini et al. (2017) was used to perform the hFE simulations. Furthermore, the statistics presented in this study were calibrated with the MSL-to-MIL relationship on segmented images at 61 µm resolution, as this is the most prevalent output that can be obtained from second-generation HR-pQCT. Figure G.12 shows the power relationship between the latter.

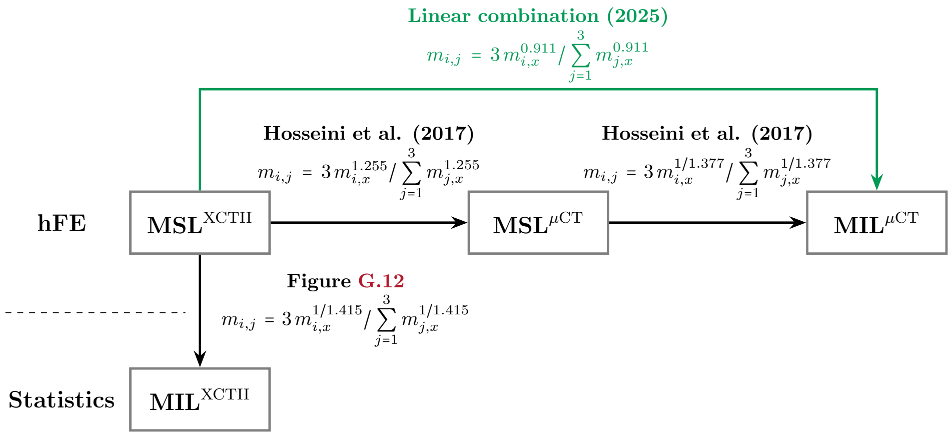

Figure G.11: Overview of the relationships between the gold standard $\mathbf{MIL}^{\mu\mathrm{CT}}$ and $\mathbf{MSL}^{\mathrm{XCTII}}$ available from the simulation pipeline.

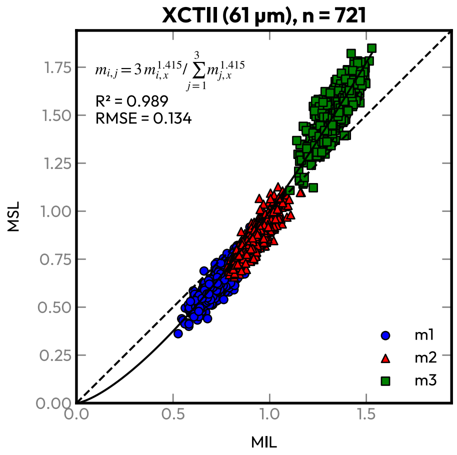

Figure G.12: Calibration of the fabric anisotropy eigenvalues of $\mathbf{MSL}^{\mathrm{XCTII}}$ against $\mathbf{MIL}^{\mathrm{XCTII}}$ on the segmented images at 61 μm resolution.

Hosseini et al. (2017) reported strong correlations between BV/TV values derived from gray-level HR-pQCT images acquired at 61 µm and those obtained from segmented μCT images at a voxel size of 16.4 µm. They observed that the bone volume fraction estimated using the BMD-based formula $\mathrm{BV/TV}^d = \frac{\mathrm{BMD}}{1200}$ provided a reasonable approximation. However, HR-pQCT systematically underestimated BV/TV compared to μCT. A linear calibration was therefore proposed. Similarly, Schenk et al. (2022) proposed a calibration equation between segmented HR-pQCT images at 61 µm and $\mathrm{BV/TV}^d$ values referenced to 16.4 µm μCT data. Figure G.13 provides a schematic representation of the aforementioned calibration process.

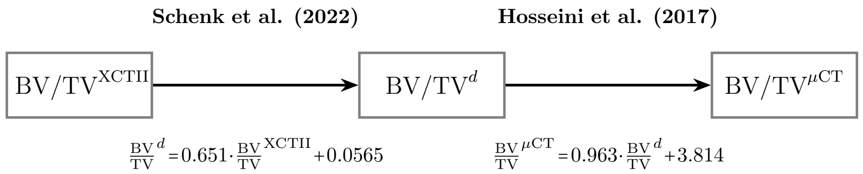

Figure G.13: Overview of the relationships between the gold standard $\mathrm{BV/TV}^{\mu\mathrm{CT}}$ and $\mathrm{BV/TV}^{\mathrm{XCTII}}$ available from the simulation pipeline.

Appendix H. Estimation of the degree of anisotropy (Tb.DA)

The Tb.DA was quantified as a volume-weighted average of the eigenvalues ratio $\frac{m_3}{m_1}$ across the trabecular mesh elements, as expressed in Equation H.1:

$$\text{Tb.DA} = \frac{\sum_{e \in E} \left(\frac{m_{3,e}}{m_{1,e}} \cdot v_e \right)}{\sum_{e \in E} v_e} \tag{H.1}$$

Where $m_3 > m_2 > m_1 (-)$, and v_e is the element volume (mm^3).

A sensitivity analysis of the ROI was performed by masking a cylindrical ROI of diameter $\varnothing = 5\,\text{mm}$ and full thickness at the center of mass, to check potential influence in the choice of the ROI. No significant differences were found. Figure H.14 shows a moderate decrease in Tb.DA with age was found. Both sexes and sites show a moderate decrease with age with a weak but statistically significant relationship.

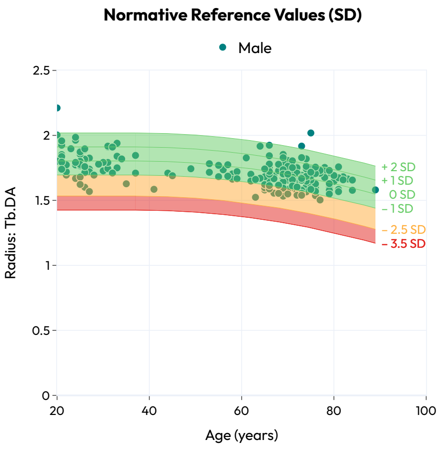

(a) Radius Tb.DA in males.

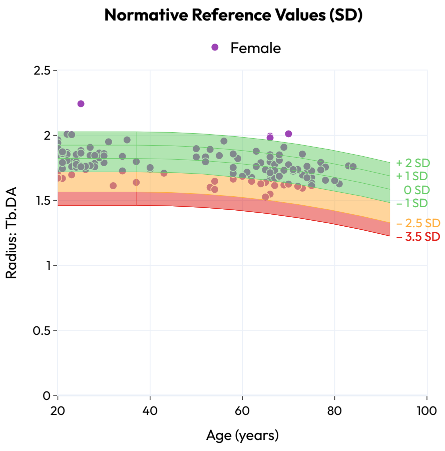

(b) Radius Tb.DA in females.

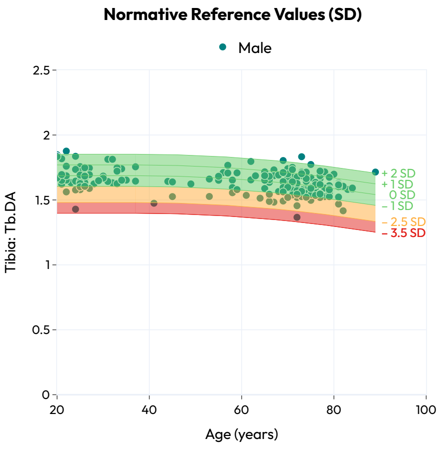

(c) Tibia Tb.DA in males.

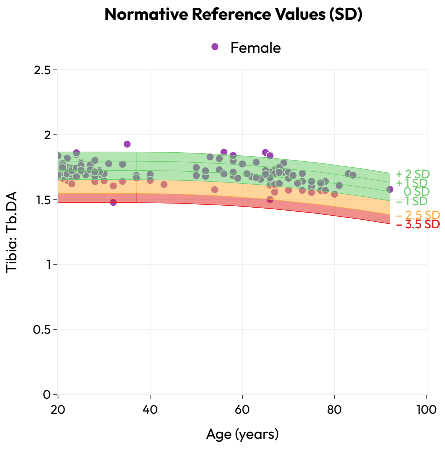

(d) Tibia Tb.DA in females.

Figure H.14: Evolution of Tb.DA over age in male (left column) and female (right column) for radius (top row) and tibia (bottom row).

Appendix I. Estimation of relative annual change and TAI

Mean and standard deviation (calculated on the younger group $G_{\leqslant 37}$)

$$\mu = \bar{y}, \quad \sigma = \sqrt{\frac{1}{n-1}\sum_{i=1}^{n}(y_i - \mu)^2} \quad \text{for } (x_i, y_i) \in G_{\leqslant 37} \tag{I.1}$$

Estimation of the short-term precision error:

$$PE_{st}{}^{1} = \sqrt{\frac{1}{n-2}\sum_{i=1}^{n}(y_i - \hat{y}_i)^2} \quad \text{for all } (x_i, y_i) \in G_{\geqslant 37} \tag{I.2}$$

With:

$$y = a \cdot x + b, \quad \hat{y}_i = a \cdot x_i + b \tag{I.3}$$

Estimation of the relative annual change in mean:

$$\frac{\Delta\mu}{\mu}/y[\%] = \frac{a}{\mu} \cdot 100 \tag{I.4}$$

Estimation of the trend assessment interval (TAI)

$$\text{TAI} = \frac{\text{PE}_{st} \cdot 1.8}{\frac{\Delta\tilde{\mu}}{\tilde{\mu}}\Big/y} \tag{I.5}$$

With:

$$\frac{\Delta\tilde{\mu}}{\tilde{\mu}}\Big/y = \frac{a_{\text{med}}}{\tilde{\mu}} \tag{I.6}$$

Where a_{med} is the median slope calculated at the 0.5 quantile.

[1] PE_{st} was calculated from the data presented by Schenk et al. (2020) [25].
